# Empirical relationship between the number of review and research articles

**DOI:** 10.1007/s11192-023-04654-0

**Published:** 2023-02-11

**Authors:** Petr Praus

**Affiliations:** 1 Department of Chemistry and Physico-Chemical Processes, VSB-Technical University of Ostrava, 17. Listopadu 15, Poruba, 708 00 Ostrava, Czech Republic; 2 Institute of Environmental Technology, CEET, VSB-Technical University of Ostrava, 17. Listopadu 15, Poruba, 708 00 Ostrava, Czech Republic

**Keywords:** Review articles, Research articles, Quadratic relationship

## Abstract

In this contribution, an empirical relationship between the number of review and research articles published per year was searched. The simple idea based on proportionality (linearity) between the numbers of both kinds of articles was expressed in terms of a quadratic relationship, in which the quadratic member can reflect negative or positive deviations from the assumed linearity. The quadratic relationship was able to describe beginning periods of research fields as well as their mature phases and to detect the unpredictably high number of review articles. It was verified by the articles published in 20 various research fields taken from the Web of Science during different time spans.

**Supplementary Information:**

The online version contains supplementary material available at 10.1007/s11192-023-04654-0.

## Introduction

Review articles play an important role in scientific publishing. They summarize the current state of specific topics and provide the critical evaluation of existing studies. Review articles can be divided into two main categories, such as narrative and systematic reviews (Gülpınar & Güçlü, 2013). A typical review should contain a critical as well as a synthetic part (Torraco, 2005). Reading review articles can be the first step to get the basic information about some scientific problem and/or to find new interesting problems and ideas, which are worth studying and investigating further. An integrative review article can be defined as an “important mode of both consolidating evidence and generating new ideas to push a field of study forward” (Elsbach & Knippenberg, 2020). The existence of review articles demonstrates a certain degree of topic developments (Bastide et al., 1989). The main features of review articles have been broadly analysed and discussed in literature (Blümel & Schniedermann, 2020; Fassin, 2021; Ho et al., 2017; Palmatier et al., 2018) including a purpose increase of the impact factors of scientific journals (Ketcham & Crawford, 2007).

In this contribution, a relationship between research and review articles was investigated. An assumption of such relationship was based on a natural and simple idea that the results of research are being published in research articles, which are consequently summarized and evaluated in review ones. And, on the other hand, a continuation of successful research is stimulated by reading review articles to see new aspects that should be further investigated. The facts given above implicate that some kind of balance can be established between the number of both kinds of articles and, hence, it should be described by some relationship.

## Data and methods

The number of review and research articles was taken from the Web of Science (WoS) (Clarivate Analytics, USA). The data from 20 various research fields of different duration were collected up to 2021, see Table 1. Only those years, in which both review and research articles were published, were used for the analysis. The data search was performed in the Web of Science Core Collection in a part “Documents”. The research topics/fields were looked up using the keywords of the names of research topics/fields.

**Table 1:** Total numbers of research and review articles in this study

Research topic/field	*N*_Rev_	*N*_Res_	*N*_Res_ + *N*_Rev_	Years	From–to
Nanotechnology	12,069	34,941	47,010	26	1996–2021
3D printing	2899	29,939	32,838	17	2005–2021
RNA vaccines	2705	10,550	13,255	31	1991–2021
Genetic engineering	7941	28,757	36,698	39	1983–2021
Microplastics	855	5931	6786	12	2010–2021
TiO_2_ photocatalysis	1509	27,101	28,610	33	1989–2021
Artificial intelligence	6481	50,524	57,005	39	1983–2021
Graphene	14,498	247,924	262,422	27	1995–2021
MXenes	475	2198	2673	8	2013–2021
CO_2_ reduction	4217	68,540	72,757	31	1991–2021
H_2_ production	8817	105,035	113,852	45	1977–2021
Pharmaceuticals in environment	2410	13,029	15,439	31	1991–2021
Powder metallurgy	379	13,236	13,615	35	1985–2021
Robotics	6780	78,997	85,777	39	1982–2021
Neuroimaging	11,113	45,651	56,764	36	1986–2021
Mars exploration	302	3073	3375	25	1997–2021
Solar cells	9051	164,773	173,824	44	1977–2021
CRISPR	4075	22,058	26,133	15	2007–2021
Global warming	5030	60,711	65,741	33	1997–2021
Ag nanoparticles	1185	52,230	53,415	24	1989–2021

The data were processed by MS Excel 2019. The statistical analysis including a non-linear regression based on the Gauss–Newton iteration procedure was performed by the QC.Expert software (TriloByte Ltd., Pardubice, Czech Republic) on the significance level α = 0.05. The outliers were detected by using the Atkinson distance, the Jackknife residuum, and diagonal elements of a projection matrix.

## Results and discussion

### Relationship between the number of review and research articles

Supposing that the above-mentioned balance exists, one can assume that the number of review articles (*N*_Rev_) published in one year should be theoretically proportional to the number of research ones (*N*_Res_) published in the same year as

1 $${N}_{\mathrm{Rev} }=k {N}_{\mathrm{Res}}$$

where *k* is the constant and *k* < 1 because the number of review articles should be lower than the number of research ones. It is possible to note that *N*_Rev_ and *N*_Res_ taken in the same years are not, in fact, synchronized in time because the review articles describe the results obtained in recent past. However, some deviations from this ideal model can be expected when the research takes a long time and review articles describe results obtained several years ago and also in an early stage of research when only a few review articles could be written about several research ones. That is why a more general quadratic relationship between *N*_Rev_ and *N*_Res_ can be suggested

2 $${N}_{\mathrm{Rev} }=a {N}_{\mathrm{Res}}^{2}+b{N}_{\mathrm{Res}}$$

where *a* and* b* are the constants (parameters). An absolute member *c* was not taken into account because if *N*_Res_ = 0 then *N*_Rev_ = 0. If the quadratic member is not significant (*a* ≈ 0) we obtain the linear relationship again.

As mentioned above, the linear member $(b{N}_{\mathrm{Res}})$ describes a new quickly developing research and the quadratic member ($a {N}_{\mathrm{Res}}^{2}$) describes a long-lasting and mature research, during which a lot of research articles were published or, on the other hand, a new developing research field at its early stage. Both kinds of articles are also associated by various publishing purposes and strategies, for example, the review articles bring more citations than the research ones (Miranda & Garcia-Carpintero, 2018). Moreover, some review articles can refer to other review ones.

The quadratic relationship (2) was tested on the number of annually published research and review articles from different research fields/topics (Table 1). The results of quadratic regression are shown in Table 2. The quadratic model was verified by the sliding window method (Rebbapragada et al., 2009) using the window of 5 years, in which the number of articles was cumulated (summed up). Two exceptions were the topics of Microplastics and MXenes with a small amount of data, for which the 3-year window was used. The regression coefficient *r* indicates how the quadratic model fits the cumulated data. Since the scientific fields were studied in different time spans, their effect on the model parameters (*a* and *b*) was tested. A weak correlation with *r* = 0.440 (*r*_crit_ = 0.423) between the quadratic parameter *a* and the time span calculated as the number of years between 2021 and the first year of publishing (Table 1) was found but there was no significant correlation for the linear parameter *b* (*r* = 0.165). The correlation shown in Figure S1 (Supplementary materials) was strongly influenced by two points A and B. After their exclusion, the correlation coefficient decreased at *r* = 0.187, which indicates insignificant correlation. Moreover, all the quadratic regressions given in Table 2 were statistically significant with high regression coefficients (*r* = 0.950 to 1.000). It can be concluded that the time span has no effect on the quadratic model parameters.

**Table 2:** Results of quadratic regression analysis of *N*_Rev_* vs. N*_Res_

Research topic/field	*a*	*b*	*r*	*N*
Nanotechnology^c^	2.02 × 10^–5^	0.0912	0.995	21
3D printing	1.46 × 10^–6^	0.0637	1.000	13
RNA vaccines^c^	4.05 × 10^–5^	0.132	0.991	26
Genetic engineering^c^	4.54 × 10^–6^	0.228	0.997	34
Microplastics^b^	1.76 × 10^–5^	0.0747	1.000	10
TiO_2_ photocatalysis^c^	3.13 × 10^–6^	0.0218	0.995	28
Artificial intelligence	3.20 × 10^–6^	0.0588	0.997	35
Graphene	2.89 × 10^–7^	0.0154	0.996	23
MXenes^b^	1.15 × 10^–4^	0.0566	1.000	6
CO_2_ reduction	3.20 × 10^–6^	0.0291	0.999	27
H_2_ production	1.01 × 10^–6^	0.0530	0.999	41
Pharmaceuticals in environment^c^	1.42 × 10^–5^	0.111	0.994	26
Powder metallurgy^c^	6.76 × 10^–6^	0.00134	0.950	30
Robotics	1.58 × 10^–6^	0.0502	0.998	35
Neuroimaging	6.39 × 10^–7^	0.241	0.995	32
Mars exploration	1.23 × 10^–4^	− 0.00357^a^	0.981	21
Solar cells^c^	4.68 × 10^–7^	0.0224	0.990	39
CRISPR	− 1.58 × 10^–6^	0.197	0.999	11
Global warming	2.47 × 10^–7^	0.0749	0.990	29
Ag nanoparticles^c^	4.16 × 10^–7^	0.0123	0.995	19

Note: ^a^Insignificant value (equals to 0), *r* is the correlation coefficient, *N* is the number of points used for the regression, ^b^3-year window was used, ^c^regression was calculated without the year 2021

### Examples of the quadratic relationship fitting

**Fig. 1:**
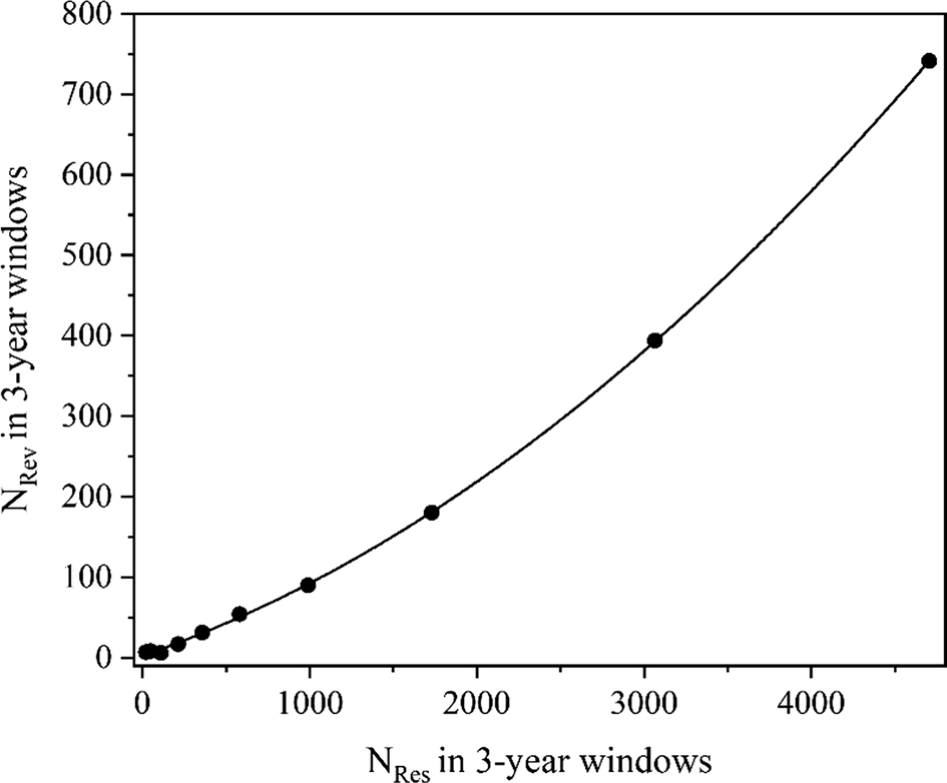
Regression graph of *N*_Rev_ vs. *N*_Res_ for the topic of Microplastics (2010–2021)

**Fig. 2:**
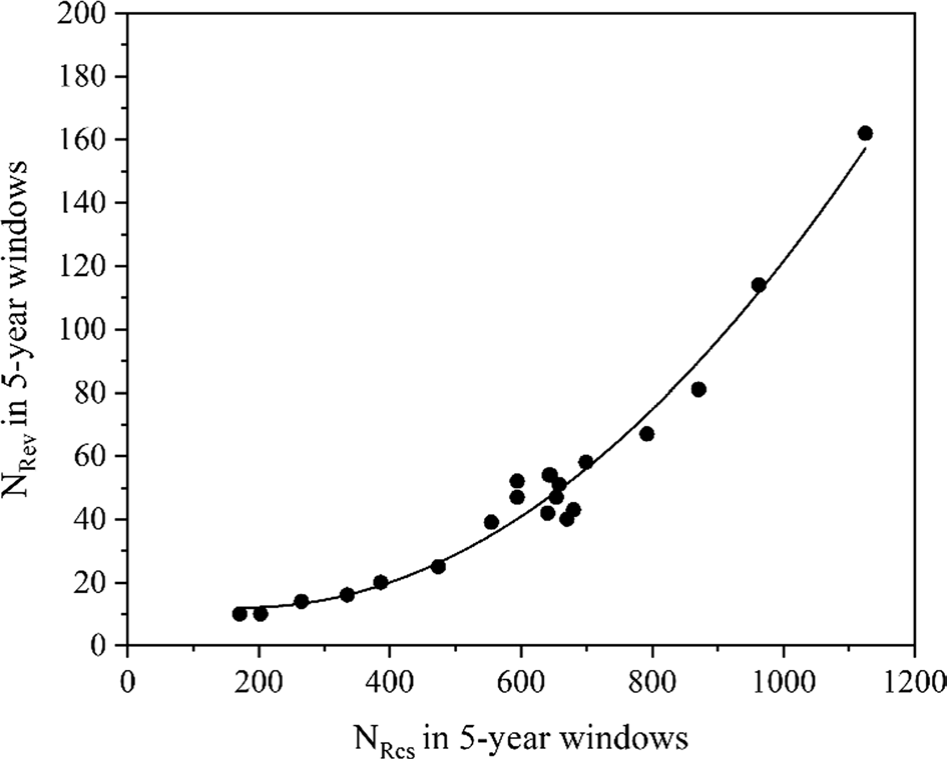
Regression graph of *N*_Rev_ vs. *N*_Res_ for the topic of Mars exploration (1997–2021)

**Fig. 3:**
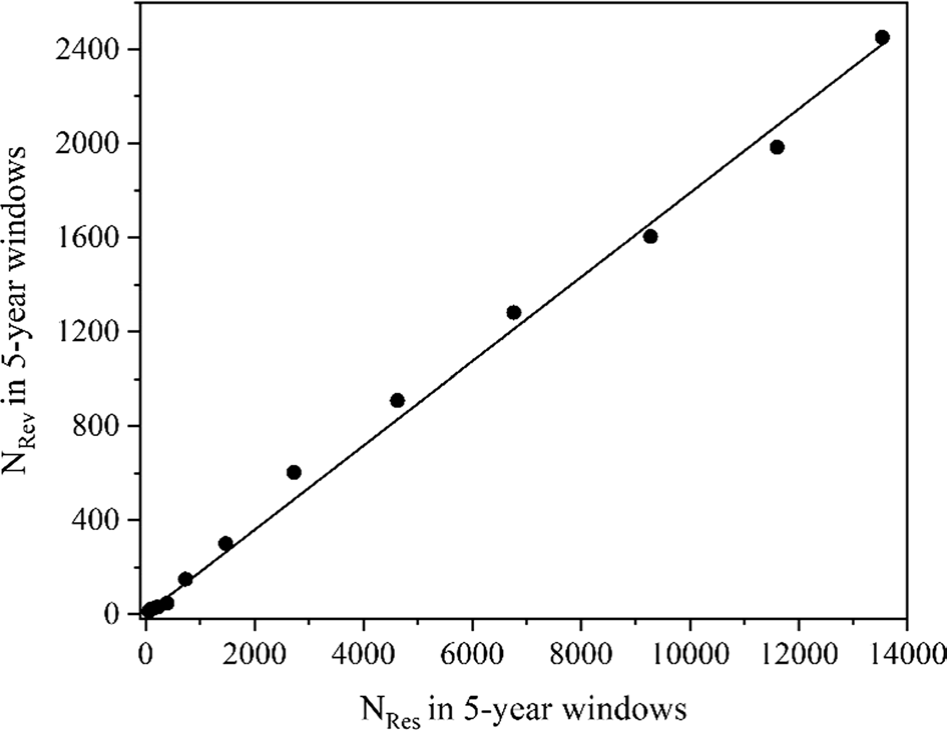
Regression graph *N*_Rev_ vs. *N*_Res_ for the topic of CRISPR (2007–2021)

An example of a good fit (*r* = 1.000) with the constants *a* = 1.76 × 10^–5^ and *b* = 0.0747 is demonstrated in Fig. 1 in the case of the topic of Microplastics, which is critical especially for the environmental contamination (Lim, 2021). Other two examples representing only quadratic or linear correlation graphs are demonstrated in Figs. 2 and 3, respectively.

The graph related to Mars exploration (Fig. 2) shows the negligible linear member and the dominating quadratic member. On the other hand, the topics such as CRISPR (Clustered Regularly Interspaced Short Palindromic Repeats) (Doudna & Charpentier, 2014), Neuroimaging, and Genetic engineering, are characterized by the relationships, in which the quadratic members are very small (*a* = − 1.58 × 10^–6^, 6.39 × 10^–7^, and 4.54 × 10^–6^ respectively) and the linear ones dominate (*b* = 0.197, 0.241, and 0.228 respectively) (Fig. 3). These nearly linear relationships indicate a stable progressive development in these new fields.

The already mentioned long-lasting research does not have to be the only reason for increasing *N*_Rev_ above the ideal linearity. Writing review articles can be considered an easier way of publishing in comparison with the demanding and expensive experimenting in a laboratory. Moreover, the review articles can easily increase author´s citations (Ho et al., 2017; Miranda & Garcia-Carpintero, 2018).

It is also shown in Table 2 that especially the *N*_Rev_ values in 2021 were higher than it could be expected according to the quadratic relationship and, hence, they were excluded from the regression as outliers. It can be caused by (i) the unpredictable increase of *N*_Res_ or, which is more probable, by (ii) restrictions due to the Covid 19 pandemic reducing research activities (Alsiri et al., 2021; Harper et al., 2020). Very likely, scientists used their “free” time and capacity to write review articles instead of working in laboratories. Here we can see an impact of the global extraordinary situation on scientific research. Similar situation is shown in the case of the field of TiO_2_ photocatalysis in 2021, see Figure S2. The year 2021 was used for the calculation of the last sliding window. Other examples of the outlying year 2021 are given in the Supplementary materials in Figures S3–S5. Using the quadratic relationships, we are able to detect situations when current research and publication activities are somehow affected.

**Fig. 4:**
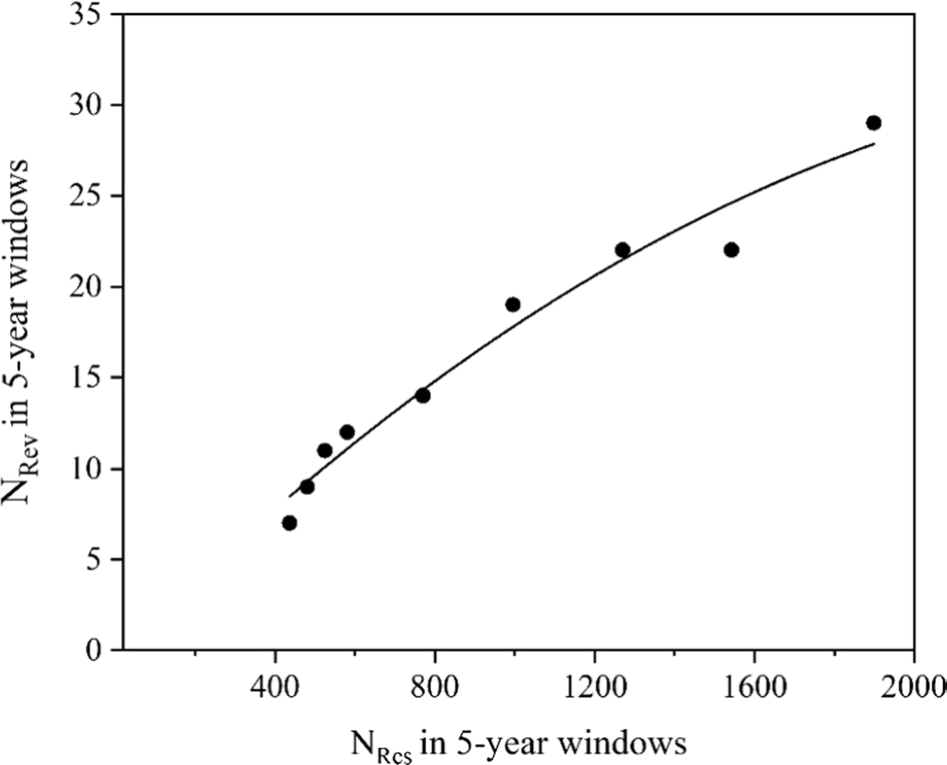
Regression graph of *N*_Rev_ vs. *N*_Res_ for the topic of Robotics (1982–1994)

Different situation concerning an early stage of research can be displayed in Fig. 4. This is the regression graph describing the beginnings of robotics when only several research articles were published about several hundreds of research ones during 1982–1994. The regression results provided significant coefficients *a* = 1.58 × 10^–6^ and *b* = 0.0502 with *r* = 0.998.

Unlike the previous examples, in this case one can see the negative deviation from linearity. The whole regression graph with the positive quadratic member concerning this robotic research until today (1982–2021) is displayed in Figure S6; the results are also shown in Table 2. It is remarkable that the Covid 19 crisis had no visible impact on this research field.

### Number of review and research articles in relation to time

**Fig. 5:**
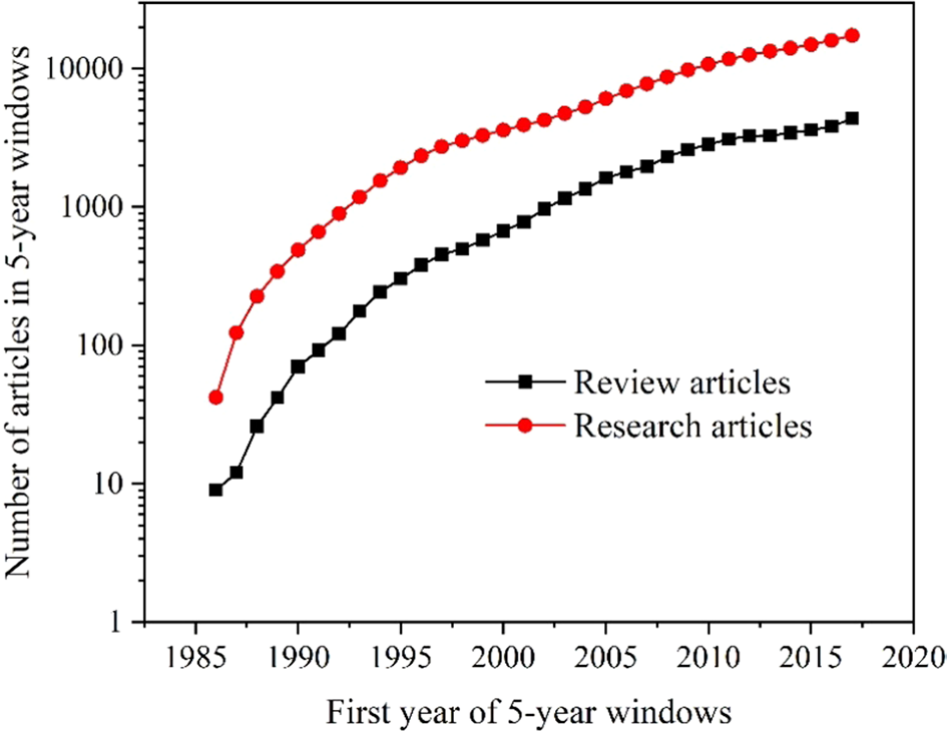
Number of articles in 5-year windows on the topic of Neuroimaging

The number of review and research articles depending on time was another part of this study. Two cases with different time courses are shown here: the topics of Neuroimaging and Graphene. In the case of Neuroimaging shown in Fig. 5 one can see the increasing number of both kinds of articles during the whole period. However, the number of articles increased steeply at the beginning of the research and then kept increasing but slowly. This was in consistency with the intensively developing scientific field described by the dominating linear member of the quadratic relationship given in Table 2 as already mentioned above (see Fig. 3).

**Fig. 6:**
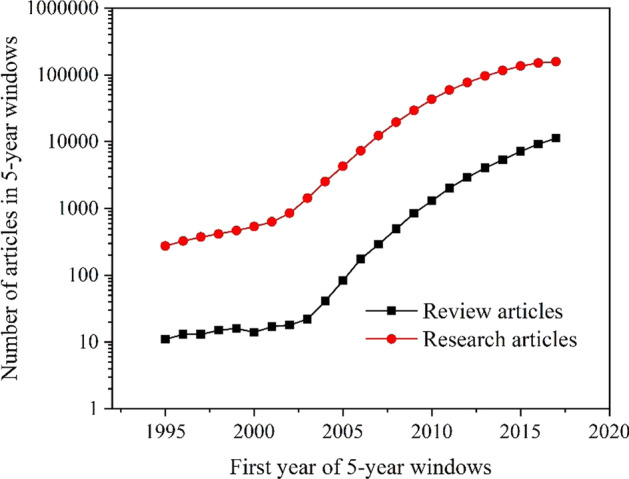
Number of articles in 5-year windows on the topic of Graphene

On the other hand, the case of Graphene was illustrated in Fig. 6 by the plots of different courses, especially at the beginning period. This scientific field was developing slowly, and the number of articles increased after several years. This behaviour is in line with the quadratic relationship with the low linear member (see Table 2) typical of the long-lasting scientific research.

## Conclusion

In this contribution, an attempt to find a relationship between the number of review and research articles was made. The basic idea was based on theoretical proportionality between the numbers of both kinds of articles published per year. The linear model describes a stably developing research. The quadratic member was added to express deviations from the linearity describing beginnings of research (negative deviation) or less intensive long-lasting research (positive deviation). The quadratic regression based on 5-year (3-year) sliding windows was calculated between the number of review articles and the number of research articles published in 20 various scientific fields.

Linear regression graphs were obtained for the fields of neuroimaging and the CRISPR technology, which have been dynamically developing. The quadratic correlation graphs were found for other research fields and were demonstrated in details for the fields of Microplastics and Mars exploration. In the case of Robotics, the early stage of this field development was demonstrated. The topics of TiO_2_ Photocatalysis, Genetic engineering, Nanotechnology, and Pharmaceuticals in environment were used to show detection of the unpredictably high number of review articles likely due to the Covid 19 pandemic in 2021. The dependence of the numbers of both kinds of articles on time was demonstrated as well. The topics of Neuroimaging and Graphene demonstrated the growth of the number of articles in line with the linear and quadratic models, respectively.

The empirical relationship allows to see the state and dynamics of research. It was tested on the data of research fields but it could be further tested on scientific journals and research institutions (universities) to find their publication strategies. Another direction of investigating can be processing review articles without other reviews referred in them.

## Supplementary Information

Below is the link to the electronic supplementary material.

Supplementary file1 (DOCX 462 KB)
