# Supplementary material for: Empirical relationship between the number of review and research articles
Source: Scientometrics. 2023 Feb 11;128(4):2201–9. doi: 10.1007/s11192-023-04654-0 (PMC9919734; doi:10.1007/s11192-023-04654-0)
Supplement: Supplementary file 1 — Supplementary file1 (DOCX 462 KB) [file 11192_2023_4654_MOESM1_ESM.docx]

**Supplementary materials**

**Empirical relationship between the number of review and research articles**

**Petr Praus^1,2*^**

^1^Department of Chemistry, VSB-Technical University of Ostrava, 17. listopadu 15, 708 00 Ostrava-Poruba, Czech Republic

^2^Institute of Environmental Technology, CEET, VSB-Technical University of Ostrava,

17. listopadu 15, 708 00 Ostrava-Poruba, Czech Republic

*****Correspondence: [petr.praus@vsb.cz](mailto:petr.praus@vsb.cz)


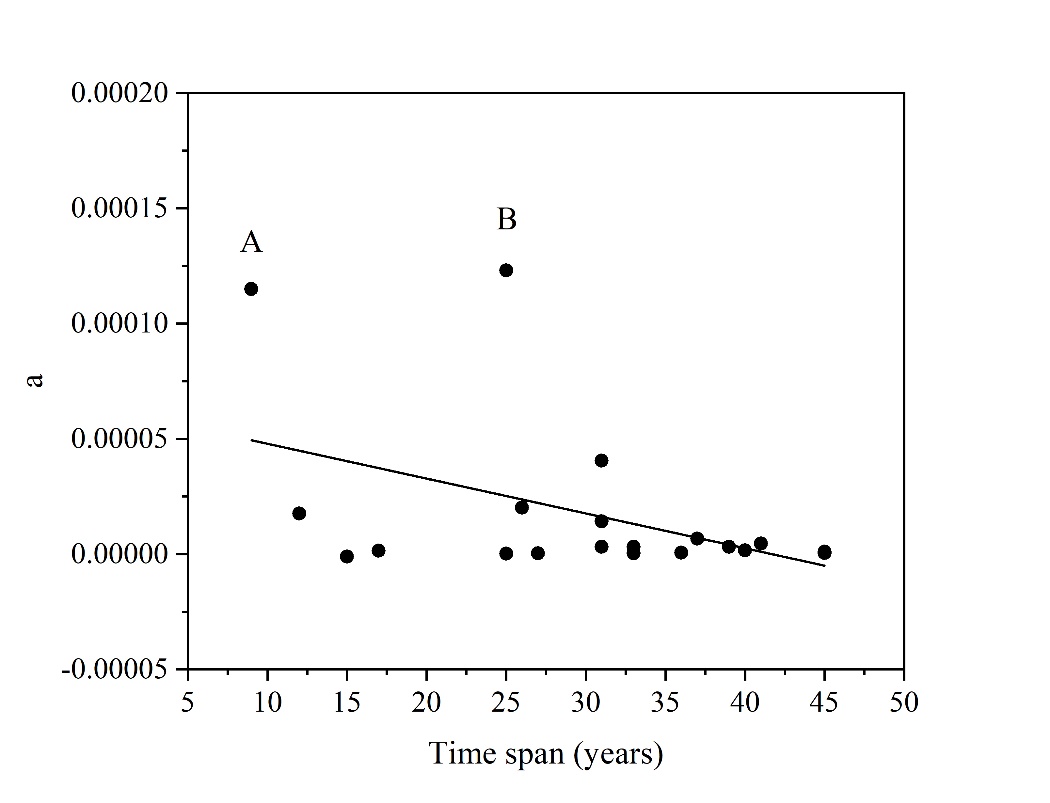


Figure S1 Regression graph of quadratic parameter a and time span. A – the topic of MXenes, B – the topic of Mars exploration.


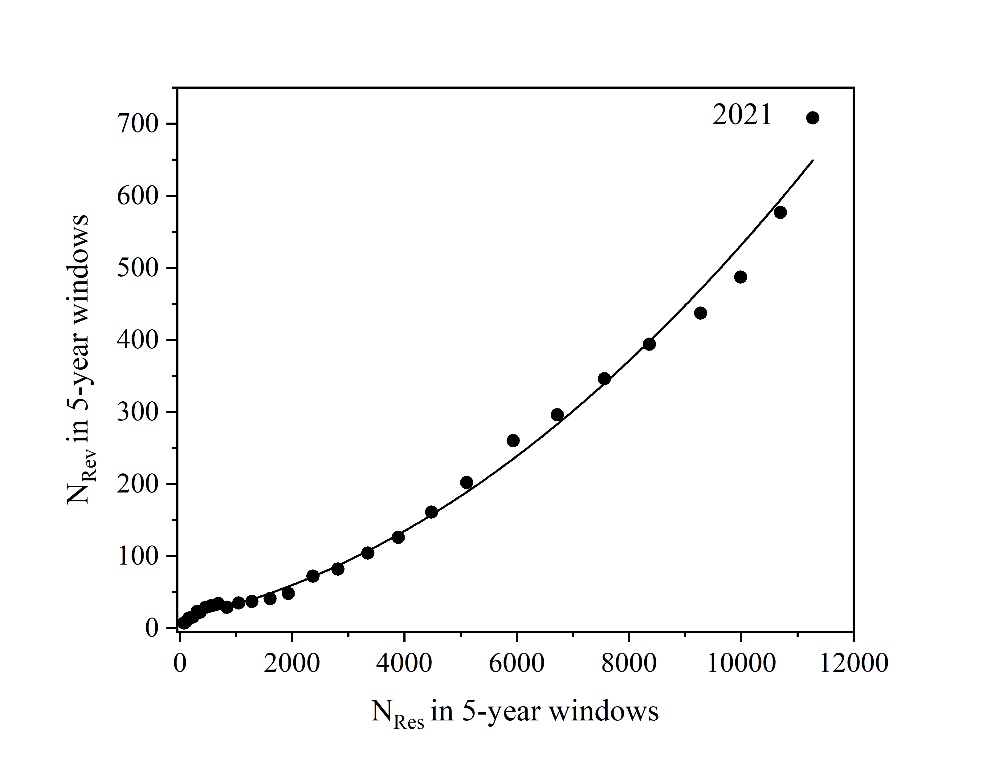


Figure S2 Regression graph of *N_Re_*_v_ vs. *N_re_*_s_ for the topic of TiO_2_ photocatalysis (1989–2021).


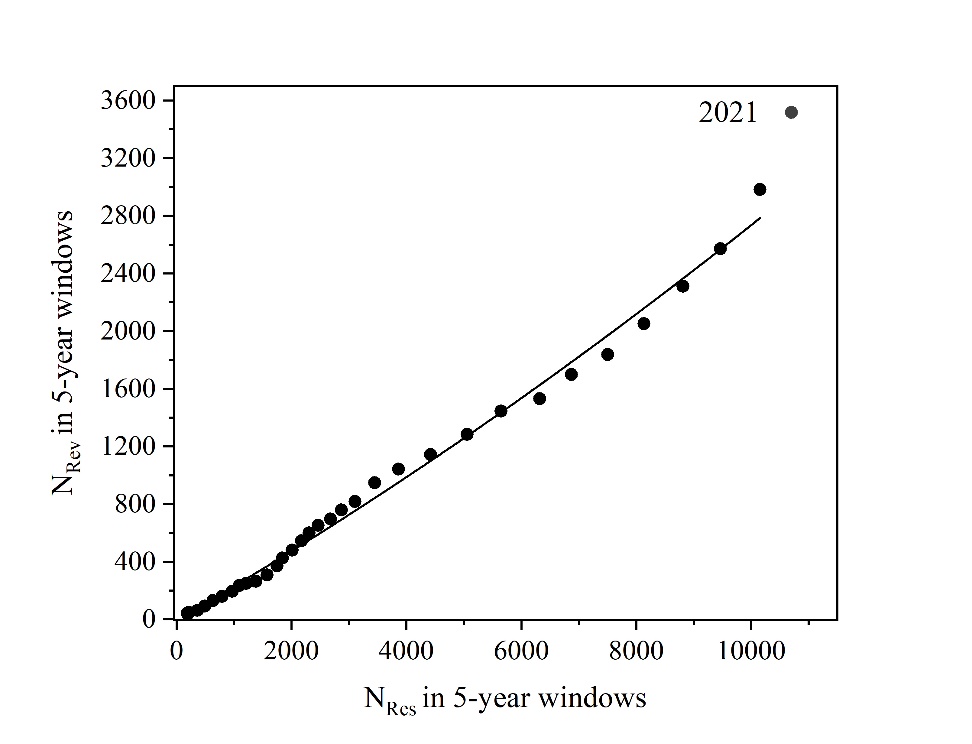


Figure S3 Regression graph of *N_Rev_* vs. *N_Re_*_s_ for the topic of Genetic engineering (1983–2021).


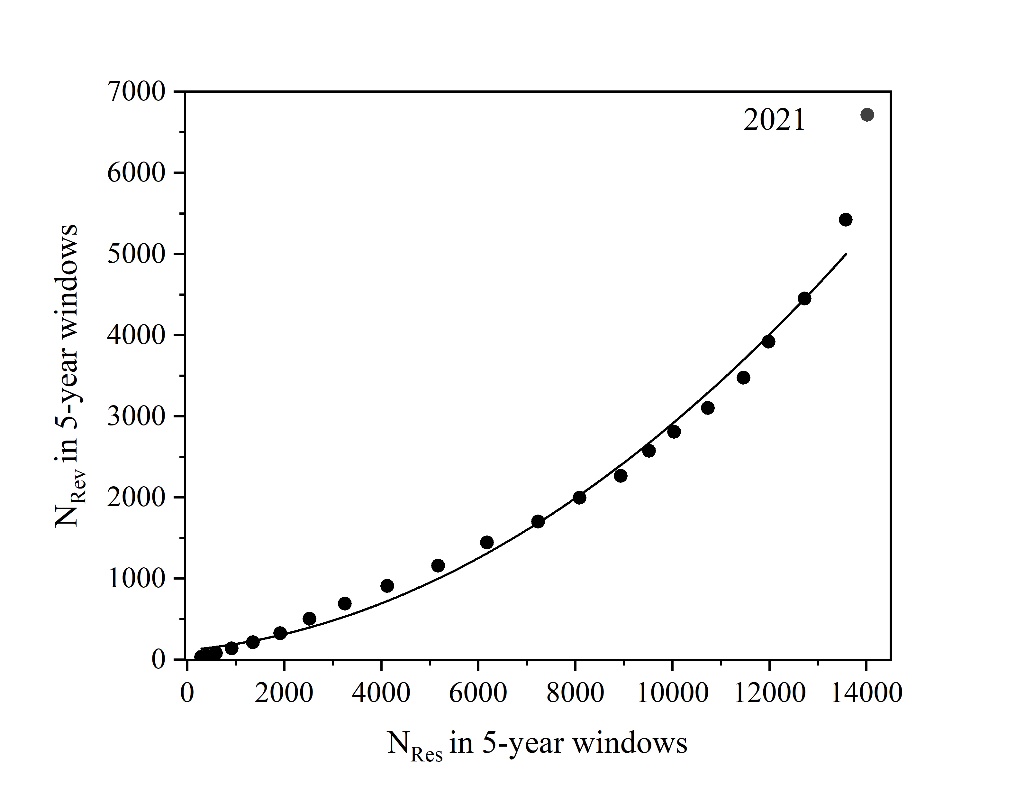


Figure S4 Regression graph of *N_Rev_* vs. *N_Re_*_s_ for the topic of Nanotechnology (1996–2021).


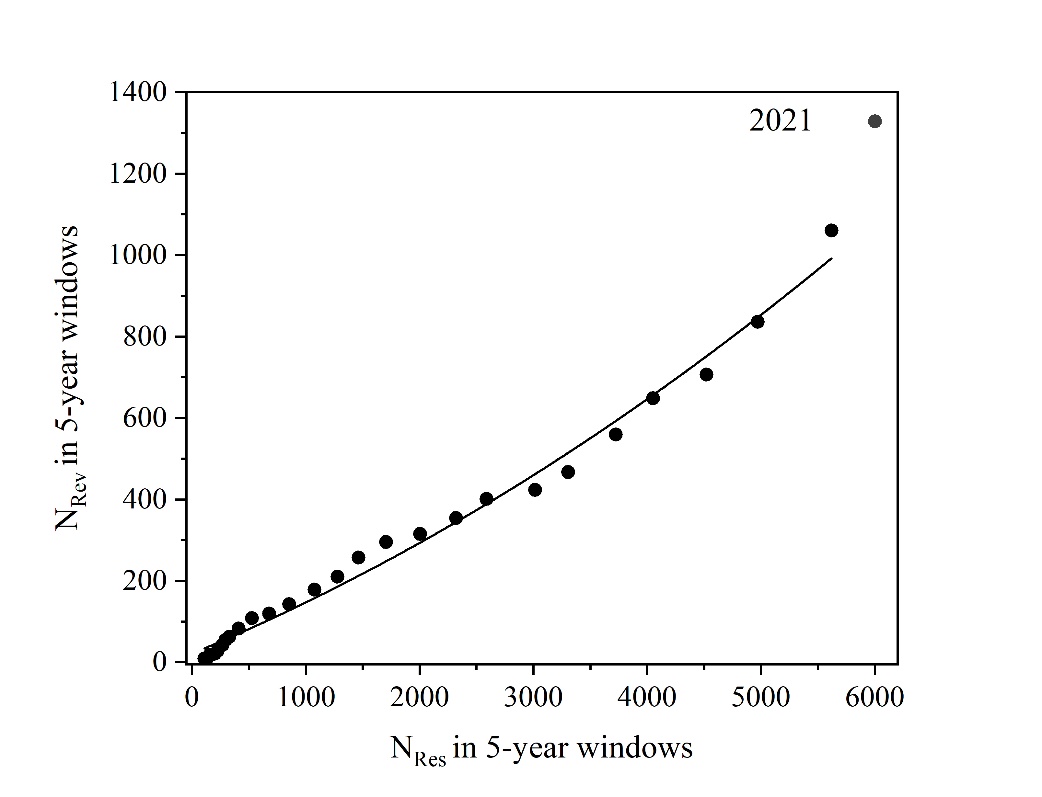


Figure S5 Regression graph of *N_Rev_* vs. *N_Re_*_s_ for the topic of Pharmaceuticals in environment (1991–2021).


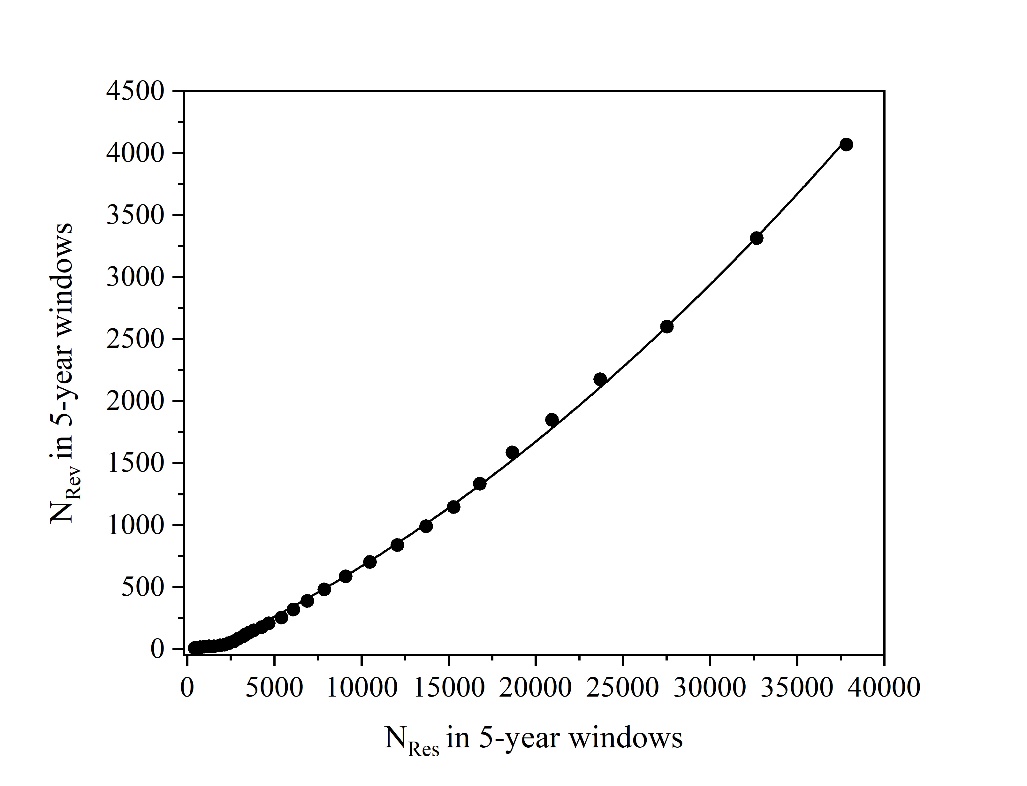


Figure S6 Regression graph of *N_Rev_* vs. *N_Re_*_s_ for the topic of Robotics (1982–2021).
